# Depression, Nutrition, and Adherence to Antiretroviral Therapy in Men Who Have Sex With Men in Manila, Philippines

**DOI:** 10.3389/fpubh.2021.644438

**Published:** 2021-09-21

**Authors:** Hend Elsayed, Cara O'Connor, Katerina Leyritana, Edsel Salvana, Sharon E. Cox

**Affiliations:** ^1^Department of Tropical Medicine and Global Health, School of Tropical Medicine and Global Health, Nagasaki University, Nagasaki, Japan; ^2^Sustained Health Initiatives of the Philippines (SHIP), Mandaluyong, Philippines; ^3^Faculty of Epidemiology and Population Health, London School of Hygiene and Tropical Medicine, London, United Kingdom; ^4^Institute of Molecular Biology and Biotechnology, National Institutes of Health, University of the Philippines, Manila, Philippines; ^5^Institute of Tropical Medicine, (NEKKEN), Nagasaki University, Nagasaki, Japan

**Keywords:** depression, adherence, anxiety, antiretroviral therapy (ART), men who have sex with men (MSM), Philippines

## Abstract

**Introduction:** Depression is the most frequently observed psychiatric disorder among HIV patients. The effect of depression on adherence among men who have sex with men (MSM) HIV patients has not been well studied in the Philippines. Depression is commonly undiagnosed and consequently untreated, which leads to a negative influence on antiretroviral therapy (ART) adherence. Other risk factors such as HIV-related stigma, self-body image satisfaction, and nutritional status are recognized as potential barriers to access HIV prevention and treatment services issues and poor adherence.

**Methods:** Hospital anxiety and depression scale (HADS) was used to screen depressive symptoms during scheduled clinic visits. ART adherence was self-reported using a visual analog scale questionnaire covering the last 30 days. Structured questionnaires were used for measuring risk factors and socio-demographic data. Anthropometry was conducted and body composition was assessed using bioelectrical impedance analysis.

**Results:** One-hundred and ninety-three participants were recruited from the SHIP clinic between 7th March and 30th September 2018, of whom, 42 (21.8%) screened positive for depression (HADS score ≥ 8) and 24 (12.4%) were non-adherent to ART (<95% of medication taken as prescribed). The most common reported reason for non-adherence was simply forgotten (18 out of 42, 42.9%). Increasing depressive symptoms were associated with non-adherence [crude odds ratio (OR) = 1.13; 95% CI: 1.02–1.26]. Social family support (SFS) and body image (BI) scores were also associated with non-adherence, but were not statistically significant in multivariable models. Factors significantly associated with depressive symptoms (but not non-adherence) included the following: using intravenous drugs, being in a relationship, anxiety, self-esteem, and stigma scores.

**Conclusions:** Increased depression symptoms, low social family support, and body image dissatisfaction may be interconnected risk factors for ART non-adherence among Filipino MSM HIV patients. Comprehensive mental health services beyond regular post-HIV testing counseling may increase adherence to ART and improve HIV treatment outcomes. Further prospective studies are needed to address the causal/reverse causal pathway between depression and non-adherence.

## Introduction

The Filipino population of men who have sex with men (MSM) is estimated to be between 200,000 and 670,000, representing 1–3% of the total adult male population (1). The group most impacted by HIV in the Philippines is MSM, representing 85% of new sexually-acquired infections since 2011. The median age of new cases is 22 years old, and more than 60% of people living with HIV in the Philippines are under 25 (2).

The efficacy of antiretroviral therapy (ART) depends on compliance with daily medication regimens. Adherence is a cornerstone to suppress viral replication and improve immunological and clinical outcomes; decrease the risk of developing ART drug resistance; and reduce the risk of transmitting HIV (3). A recent surveillance study in the Philippines showed that 10.3% of patients experienced treatment failure on their first-line regimens, possibly due to incomplete adherence to ART (4). Also, a regional cohort study TREAT-Asia (Therapeutic Research, Education, and AIDS Training in Asia) found that out of 1,316 participants, 26% self-reported suboptimal adherence levels (<95%) during their first 6 months of treatment (5). More generally, one out of four ART users may fail to adhere to medication for reasons which can be categorized as relating to patient and family, medication, health delivery system, caregiver, and social/environmental factors (6).

People living with HIV may face many challenges including depression, low social and family support (SFS), widespread stigma, changes in nutritional status, and barriers to accessing mental and psychological care which may have a negative effect on ART adherence level (7). People living with HIV are more likely to be depressed compared with those who are HIV negative (8), and MSM are three times more at risk of depression compared with the general population of men (9). Depression has a severe negative impact on the quality of life (QOL), which can affect patient behaviors including medication adherence (10). Depression in people living with HIV could be triggered by stress, stigma, difficult life events, and side effects of medications (11, 12). A crosssectional study in Malawi reported depression to be associated with a 1.55 fold greater risk of being non-adherent to ART (13). A study in Vietnam found that several clinical and social factors were associated with depression among PLHIV, and these factors included having a lower number of CD4 cells at the start of ART, receiving ART in the clinic without HIV counseling and testing (HCT) services, having a physical health problem, and experiencing discrimination (14).

Stigma is another barrier for HIV patients. UNAIDS reported in October 2017 that across 19 countries, one in five persons living with HIV avoided going to health centers because they feared stigma related to their HIV status (13). PLHIV are often non-adherent to their ART to avoid being seen taking pills and avoid being perceived as HIV-positive (15). People living with HIV may experience social isolation, lose employment, and family and friends as major sources of support (3). Previous studies in Thailand, France, and South Africa have found a positive association between SFS and ART adherence (15–17).

Persons living with HIV are highly susceptible to malnutrition due to inadequate dietary intake, appetite loss, nutrition losses, metabolic changes, and increased requirements for both macro and micronutrients (18). Those who are undernourished [defined as body mass index (BMI) < 18.5 kg/m] are 10 times more likely to be non-adherent to ART (19). In a prospective cohort study in Haiti, receiving food assistance and subsequent improved nutritional status was associated with improved adherence to ART (18). Up to 60% of MSM HIV infected individuals experience moderate to severe changes in body fat composition (20, 21). It has been previously reported that MSM living with HIV are more likely to experience high levels of body dissatisfaction compared with heterosexual persons living with HIV (22).

A lack of data exists on depression in Filipino MSM persons living with HIV. The primary objective of this study was to assess the prevalence of depression and its association with ART adherence and with other risk factors, such as stigma, low self-esteem, alcohol abuse, body image, nutritional status, and low social and family support among Filipino MSM. We hypothesized that these risk factors would be correlated and associated with both depression and adherence.

## Methods

### Study Design

This was a nested crosssectional study within an ongoing single-arm, mobile health (mHealth) intervention study which used a self-reported questionnaire for adherence to assess the impact of the intervention on ART adherence.

### Study Population

The inclusion criteria for participation in the nested study were as follows: (1) adult PLHIV (age 18 years); (2) male subjects who self-defined as having sex with males; (3) taking ART in the sustained health initiatives of the Philippines (SHIP) clinic in Manila; (4) enrolled into an existing, single-arm trial study: the Connect for Life Mobile Phone Adherence Demonstration Project (CFL) at the SHIP Clinic; (5) able to understand written and spoken English. The exclusion criteria were the following: (1) receiving primary HIV care at a facility other than SHIP; (2) late-stage HIV and/or hospitalized; (3) previous psychiatric disease before diagnosis of HIV or any significant clinical neurological disorder such as stroke or cerebral palsy.

### Study Site

Sustained Health Initiatives of the Philippines Clinic is a low-cost, private facility in Metro Manila, a city of just under 13 million people in the predominantly Catholic country of the Philippines. SHIP Clinic provides HIV primary care and wrap-around services to approximately 900 patients as of April 2021. Between 2012 and 2018, SHIP was a satellite partner clinic of the STI/AIDS Guidance Intervention and Prevention Unit at the Philippine General Hospital (PGH-SAGIP), the largest hospital in the country. Approximately 98% of clients of SHIP are MSM, with an average age of 30 years at initial consultation. Most are employed full- or part-time. The patients come from all regions of Metro Manila and some live outside of Metro Manila in other provinces.

### Study Enrolment Procedures

Participants in the CFL study were approached to participate in this nested study by a member of the study team during a routine clinic visit and written informed consent was obtained.

### Data Collection

Data were collected using a self-reported questionnaire, including standardized data collection tools as described below, designed, and implemented through Open Data Kit (ODK) using an electronic tablet during a routine clinic visit. Trained clinical research assistants were available to answer any queries from participants while completing the questionnaire.

Antiretroviral therapy adherence data was extracted from CFL study records in which adherence was assessed using a self-administered “Visual Analog scale” question of the percentage of doses taken in the previous 30 days at four-time points, namely, baseline [Week 0 (within 60 days from Screening)] and 12, 24, and 48 weeks. Participants were defined as adherent at each time point if the participant reported 95% of medications taken in the prior 30 days.

CD4 data was extracted from participant records. In the study site, ART initiation was recommended for all patients with an AIDS-defining illness (Treat all policy), or when CD4 count is below 500. CD4 count is performed every 6 months and the viral load test is performed annually.

Depression/anxiety was assessed using the hospital anxiety and depression scale (HADS), a self-administrated questionnaire consisting of 14-items, seven questions assessing depressive symptoms and seven assessing anxiety. Each item contains four response options (from 0 to 3). The presence of anxiety or depressive symptoms was defined as a score of HADS ≥ 8 for each (23–25). The HADS questionnaire had been validated in several languages and countries, including the Philippines (26), and in general practice and community settings (26, 27).

Alcohol abuse was measured using the alcohol use disorder identification test (AUDIT) and is recommended for use in the Philippines (28). The AUDIT is a 10-item, self-rating questionnaire that assesses hazardous drinking, dependence symptoms, and harmful alcohol use (29). The presence of alcohol-related social problems and medical complications was defined as an AUDIT score ≥ 8 (29, 30).

Social and family support was measured using the multidimensional scale of perceived social support (MSPSS) which measures the extent to which an individual perceives social support from three different sides: family, friends, and a significant other (31, 32). The scale is 12 items, with each item using a 7-point Likert scale (32). MSPSS has been validated for use in lower-middle-income countries (33–36). High levels of perceived support were defined as total score 69–84; moderate 49–68; and low 12–48 (32, 36).

Stigma was measured using the Berger scale, designed to determine stigma in HIV patients. It includes 40 items rated on a 5-point scale from (37) assessing personalized stigma, disclosure concerns, negative self-image, and concern with the public attitude. Higher scores indicate more stigma (37, 38).

Self-esteem was measured using Rosenberg self-esteem scale (RSES). It is a validated tool used to assess positive and negative feelings of a patient to his own self (39, 40). It consists of 10 questions with a 4-point Likert scale ranging from very negative (1) to very positive (4) (41, 42). Higher scores indicate higher self-esteem.

Self-body image was measured by the body image quality of life inventory (BIQLI), developed to monitor both positive and negative effects of body image on a the psychosocial QOL of a patient, which includes beliefs and emotions (43). This scale has 19 items, each using a 5-point Likert scale. BIQLI assesses the specific domains of day-to-day emotions, self-esteem, sexuality, social interest/avoidance, interpersonal relations, eating and exercise, grooming habits, and general life satisfaction (44, 45). Higher scores indicate high satisfaction with body image (44).

Anthropometry was conducted by trained clinical research assistants at the time of the interview. Height was measured using a stadiometer (model Seca 213) to the nearest 0.1 cm. Mid upper arm circumference (MUAC) was measured using a non-elastic plastic tape at the midway between the olecranon and acromion process on the upper left arm. Body composition (body fat%, lean body mass, and visceral fat) was measured using bioelectrical impedance analysis (BIA) (model Tanita MC-780MA).

### Ethics Statement

The primary intervention study (CFL) was approved by the University of the Philippines Manila (reference number UPMREB 2016-265-01) and London School of Hygiene and Tropical Medicine (reference number 11631). This nested crosssectional study was approved by Nagasaki University, School of Tropical Medicine, and Global Health (reference number 42) and the University of the Philippines Manila (reference number UPMREB 2017-453-01).

### Data Analysis

Adherence data were used from the same time point as the current data collection. Data analysis was conducted using Stata 14.0. The proportion of study participants with depression or anxiety (HADS scores ≥ 8) are reported as percentages with corresponding 95% confidence intervals. Logistic regression was used to investigate associations between depressive or anxiety symptoms and being adherent/non-adherent as a binary outcome. A *p*-value of 0.05 was considered as statistically significant. Multivariable models were developed using a forward stepwise approach from factors associated in univariable analyses significant at *p* < 0.05 and models compared using likelihood ratio (LR) tests.

## Results

**Figure 1:**
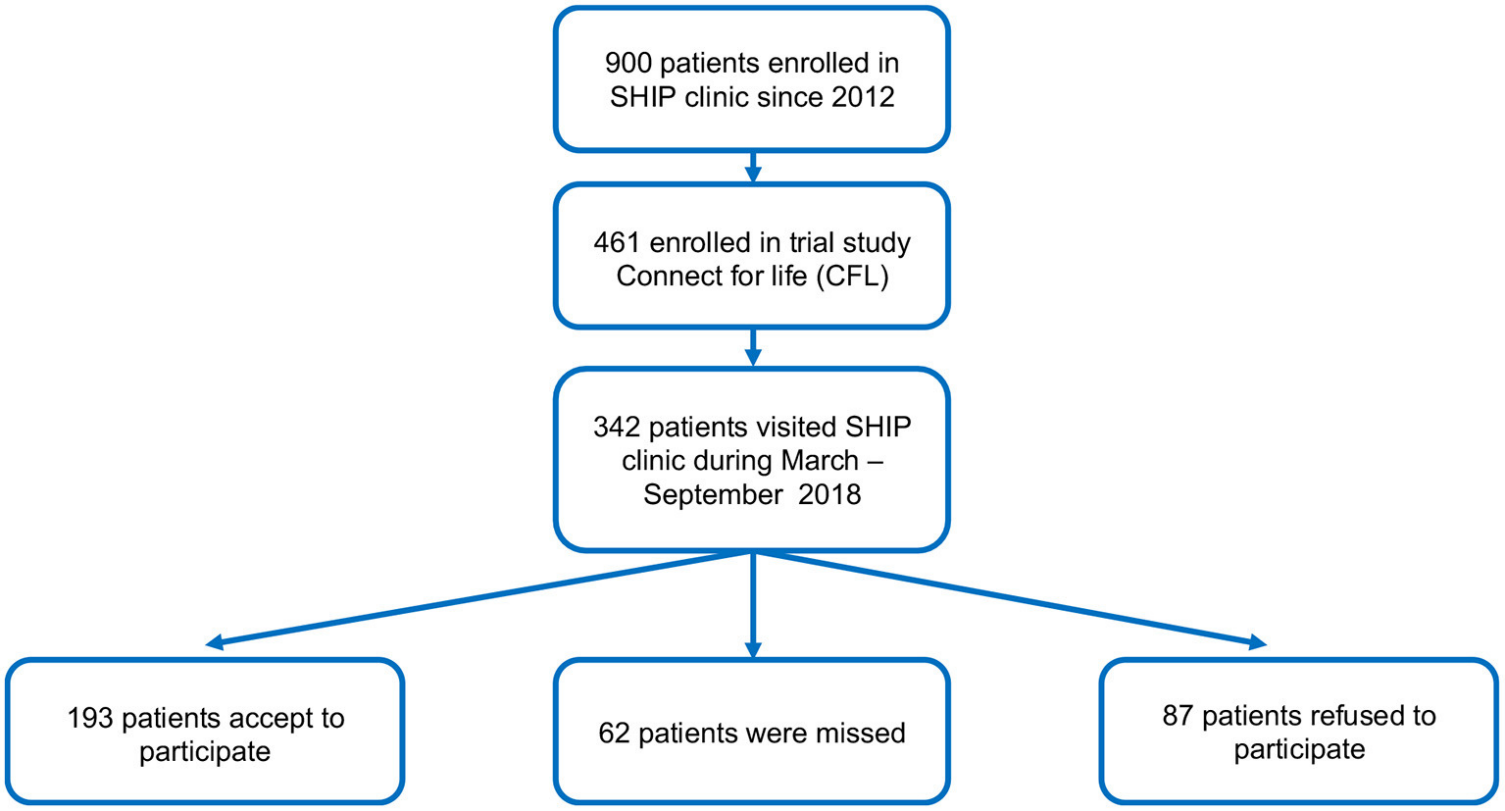
Study participant flow.

Figure 1 shows the flow of persons potentially eligible and those enrolled in the current study. Out of 342 participants in the CFL study with routine clinic visits during the nested study enrolment period, 193 Filipino MSM were enrolled between 7th March and 30th September 2018 (Figure 1). The characteristics of our study participants are shown in Table 1. The mean age was 31.95 years (range 22–53, SD 5.90). Only 48 (24.9%) reported being in a relationship. Most participants, 163 (84.5%) were highly educated at college or higher level. Fifty-nine (30.6%) had disclosed their HIV status and 91 (47.15%) reported using condoms, with consistent condom use in 77 participants (39.0%). The proportion reporting ever using IV drugs was 29 (15.0%).

**Table 1:** Demographic characteristics.

**Demographic factor**	**Freq**.	**%**
**Total**	193	
**Age**		
Mean (SD)	31.9 (5.90)	–
Median (Range)	31 (22–53)	–
**Timepoint**		
First week	2	1.04%
12 weeks	4	2.07%
24 weeks	47	24.35%
48 weeks	140	72.54%
**Sexual orientation**		
Homosexual	123	63.73%
Bisexual	64	33.16%
Not reported	6	3.11%
**Education level**		
College and higher	163	84.46%
High school and vocational	9	4.66%
Not reported	21	10.88%
**Employment**		
Employed	171	88.60%
Unemployed	22	11.40%
**Relationship status**		
No relationship	114	59.07%
In relationship	48	24.87%
Not reported	31	16.06%
**Number of partners**		
Mean (SD)	3.1 (9.00)	–
**Disclosure status**¶		
Disclosed	59	30.57%
Not disclosed	97	50.26%
Not reported	37	19.17%
**Civil status**		
Single	181	93.78%
Married	7	3.63%
Not reported	5	2.59%
**Sex desire**		
Men	162	83.94%
Both	29	15.03%
Not reported	2	1.03%
**Active sexually**		
Yes	94	48.70%
No	91	47.15%
Not reported	8	4.15%
**Using condom**		
Yes	91	47.15%
No	94	48.70%
Not reported	8	4.15%
**Frequency using condom**		
Always/most of the time	77	39.90%
Sometimes/never	110	56.99%
Not reported	6	3.11%
**Participate in commercial sex**		
No	179	92.75%
Yes	11	5.70%
Not reported	3	1.55%
**Using iv drugs**		
No	160	82.90%
Yes	29	15.03%
Not reported	4	2.07%

¶ *Disclosure status: the process of revealing the HIV status of a person*.

The prevalence of non-adherence was 12.4% (24/193) with 125 (65%) of those adherent reporting 100% adherence in the previous 30 days. The prevalence of depression was 21.8% (42/193) and anxiety 37.3% (72/193) (Table 2). The prevalence of poor SFS, poor self-esteem, and potential alcohol misuse was 34.7, 46.1, and 15.0%, respectively (Table 2).

**Table 2:** Prevalence and associations between depression, anxiety, self-esteem alcohol misuse, and social family support and ART adherence.

	**Adherent *N* = 169 (87.6%)**	**Non-adherent *N* = 24 (12.4%)**	**X^**2**^ *P*-value^a^**	**Total *N* = 193**	**OR (95% CI)**
**Depression** ***N*** **(%)**					
Not depressed (HADS score <8)	135 (89.4%)	16 (10.60%)	0.142	151 (78.2%)	1.98 (0.78–5.02)
Depressed (HADS score ≥ 8)	34 (80.95%)	8 (19.05%)		42 (21.8%)	
**Anxiety** ***N*** **(%)**					
Not anxious (HADS score <8)^b^	108 (89.26%)	13 (10.74%)	0.356	121 (62.6%)	1.5 (0.63–3.55)
Anxious (HADS score ≥ 8)	61 (84.72%)	11 (15.28%)		72 (37.3%)	
**Self-esteem** ***N*** **(%)**					
Good self-esteem (RSES ≥ 21)^c^	94 (90.38%)	10 (9.62%)	0.199	104 (53.9%)	1.75 (0.74–4.17)
Poor self-esteem (RSES <21)	75 (84.27%)	14 (15.73%)		89 (46.1%)	
**Alcohol behavior** ***N*** **(%)**					
No alcohol problem (AUDIT <8)^d^	143 (87.20%)	21 (12.80%)	0.711	164 (84.9%)	0.79 (0.22–2.83)
Alcohol problem (AUDIT ≥ 8)	26 (89.66%)	3 (10.34%)		29 (15%)	
**Social and family support** ***N*** **(%)**					
Good/Moderate support (MSPSS ≥ 49)^e^	115 (91.27%)	11 (8.73%)	0.032*	126 (65.2%)	2.52 (1.06–5.98)
Less support (MSPSS <49)	54 (80.60%)	13 (19.4%)		67 (34.7%)	

a *X^2^, chi-square significance test*,

b *HADS, Hospital Anxiety and Depression Scale;*

c *RSES, Rosenberg Self-Esteem Scale;*

d *AUDIT, Alcohol Use Identifier Test;*

e *MSPSS, Multidimensional Scale of Perceived Social Support. Bold values indicate that p-value < 0.05*.

No associations between age, timepoint, CD4 count, or demographic variables (as per those in Table 1) with adherence were observed (Supplementary Table 1).

### Effects of Psychological, Social, and Nutrition Factors on Non-adherence

For exposures with predefined cut-offs and analyzed as a binary exposure, only high/moderate SFS compared with low SFS was significantly associated with odds of non-adherence (OR = 2.52; 95% CI:1.06–5.98) (Table 2). Although there were more non-adherent participants amongst those who were depressed (19.05%) compared with non-depressed (10.60%), this did not reach statistical significance (Table 2). When analyzed as continuous scores, there was good evidence of an association between increased depression symptom score and non-adherence (OR:1.13; 95% CI 1.02–1.26) as well as poorer self-body image score [OR: 1.04; 95% CI (1.01–1.08)] and decreased perceived SFS (OR = 1.03; 95% CI:1.00–1.07) (Table 3). While higher BMI had weak evidence of association (*p* = 0.0818) with non-adherence, there was no evidence for an association between nutritional status assessed by MUAC (*p* = 0.4935) or with measures of body composition (Table 3).

**Table 3:** Social, psychological, and nutrition factors and ART non-adherence as continuous measure.

**Risk factor**	**Adherent *N* = 169**	**Non-adherent *N* = 24**	**Crude OR (95%CI)**
**Social and psychological factors**			
**Age (years)**			0.97 (0.90–1.05)
Mean (95% CI)	32.05 (31.15–32.96)	31.20 (28.94–33.47)	
**Depression HADS score**			1.13 (1.02–1.26)
Mean (95% CI)	4.46 (3.92–5.00)	6.42 (4.54–8.30)	
**Anxiety HADS score**			1.06 (0.97–1.16)
Mean (95% CI)	6.62 (5.97–7.28)	7.79 (5.73–9.86)	
**Self-esteem score**			1.06 (0.99–1.14)
Mean (95% CI)	21.31 (20.43–22.18)	19.21 (16.51–21.90)	
**Social and family support score**			1.03 (1.00–1.07)
Mean (95% CI)	52.79 (50.87–54.70)	46.33 (40.18–52.49)	
**Alcohol misuse score**			1.01 (0.93–1.11)
Mean (95% CI)	3.63 (2.89–4.36)	3.92 (2.59–5.24)	
**Stigma score**			0.99 (0.97–1.00)
Mean (95% CI)	116.88 (25.81)	108.29 (28.06)	
**BIQLI score**			1.04 (1.01–1.08)
Mean (95% CI)	70.17 (68.47–71.86)	62.88 (57.49–70.25)	
***Nutritional factors***			
**BMI (kg/m** ^**2**^ **)**			1.13 (0.98–1.29)
Mean (95% CI)	23.71 (23.22–24.20)	24.92 (23.67–2618)	
**MUAC (cm)**			1.04 (0.93–1.16)
Mean (95% CI)	30.03 (29.42–30.62)	30.62 (28.92–32.32)	
***Body compositions*** ^a^			
**Waist/hip ratio**			3.89 (0.00–3279.80)
Mean (95% CI)	0.91 (0.90–0.92)	0.92 (0.89–0.94)	
**Total body fat (%)**			1.02 (0.95–1.10)
Mean (95% CI)	17.42 (16.52–18.31)	18.04 (15.37–20.70)	
**Total lean body mass (%)**			1.00 (0.96–1.05)
Mean (95% CI)	77.54 (76.03–79.05)	77.83 (75.30–80.36)	
**Visceral fat (%)**			1.05 (0.90–1.22)
Mean (95% CI)	5.02 (4.59–5.44)	5.38 (4.12–6.63)	
**Central fat mass (kg)**			1.01 (0.96–1.08)
Mean (95% CI)	19.54 (18.43–20.64)	20.25 (16.81–23.69	
***Clinical/HIV associated*** ^b^			
**Cd4 count (cells per** **μI)**			1.005 (0.99–1.02)
Mean (95% CI)2	493.21 (456.87–529.55)	529.73 (423.86–634.89)	

*Measurement of nutritional factors was missing for 1 person for BMI*,

a *for MUAC and 2 for body composition measurements—all in the adherent group;*

b *Four patients did not have data for CD4 count—all in adherent group*.

We then investigated if any of the assessed psychosocial and nutritional measures were also associated with depression and could therefore be possible confounders of the association between HADS score and non-adherence. Higher HADs score for anxiety symptoms, higher stigma, lower self-esteem, lower perceived social family support, and lower body image scores were all significantly associated with depression as well as lower measures of body fat (Supplementary Table 2).

When adjusting for SFS or body image, the association between depression score and non-adherence, was not statistically significant (Table 4). However, when comparing the crude and adjusted models, no evidence of statistical difference between the models was observed (LRT *p*-values 0.27 and 0.23; Table 4).

**Table 4:** Comparison of models of association between depression and non-adherence with and without adjustment for potential confounders.

**Model**	**OR for HADS score**	**Wald test *p*-value**	**LRT *p*-value comparing models**
Depression score Crude association	1.13 (1.02–1.26)	**0.0159**	NA
Depression score + social/family support	1.09 (0.96–1.24)	0.179	0.27^a^
Depression score + body image	1.08 (0.94–1.24)	0.255	0.23^b^

a *Comparing model including depression score and SFS score with depression score only*.

b *Comparing model including depression score and BI score with depression score only. Bold values indicate that p-value < 0.05*.

## Discussion

To our knowledge, this is the first study in Filipino MSM living with HIV to assess the prevalence of depressive symptoms with other risk factors, which was observed to be 21.8%, that has provided evidence to suggest an independent association between non-adherence to ART and depressive symptoms, perceived lower social family support, and poorer self-body image. The prevalence of depressive symptoms in this study was lower than that observed in a recent study of Chinese MSM living with HIV (36%, 95% CI 1.03–1.05) (46) which also used HADS. This difference may result from the higher educational status and lower prevalence of drug use in our population compared with the Chinese study population (46). In another study in Tanzania, the prevalence of depression among MSM persons living with HIV was also higher at 46.3% (47), possibly explained by the study in Tanzania using the Patient health questionnaire (PHQ) with cut-off point 4, which in a Swedish primary care population diagnosed 30% more as depressed compared with HADS (≥8) (48).

The prevalence of non-adherent persons in this study was 12.4%, lower than many other previous studies, but is perhaps not unsurprising in this study in which all the participants were recently enrolled into a mobile intervention study to improve ART adherence and were relatively young, urban, and highly educated. Furthermore, adherence may be higher in the newer easier to take ARV regimens, and or the finding is similar to a recent study in the urban Filipinos (4). In Cameroon, the prevalence of ART non-adherence in 300 HIV infected persons was 47.3% (49), defined using the Morisky scale ≥ 1. While in 418 Ethiopians with HIV, the prevalence of self-reported ART non-adherence (<95% of prescribed doses) over the previous 7 days was 53.4% (50). Differences may result from different methods of assessing and defining non-adherence, in study population characteristics (e.g., level of education; 69% in Ethiopia reported no education), and clinic settings, which may affect communication and relationships with health care providers (51, 52).

In this crosssectional study, we observed that an increase in 1 point of the HADS depression symptom score was associated with a 13% increase in the odds of reporting non-adherence, although the association with depression using HADS ≥ 8 did not reach statistical significance. This is consistent with previous findings in other populations. In a prospective study in 400 Ethiopians living with HIV, depression measured using a 13-item scale, widely used in the HIV/AIDS literature (≥10 defined as depressed), was significantly associated with non-adherence assessed using a self-reported scale over the last 7 days (OR 2.13; 95% CI: 1.18–3.81) (53). Two crosssectional studies, one in Thailand in 379 participants with depression assessed using the Beck depression inventory II (≥14 defined as depressed) was associated with non-adherence measured by self-reported scale over 30 days (<95%) (OR 4.68; 95% CI: 2.78–7.88) (54), and in Cameron in 300 participants, depression using the patient health question-9 (≥10 defined as depressed) was associated with adherence assessed by Morisky scale (≥1) (OR 5.04; CI: 2.84–8.97) (49).

Social family support and body image dissatisfaction were also strongly associated with non-adherence. This is consistent with the findings in the above Ethiopian study in which participants with higher perceived social family support were 1.82 times more likely to be adherent to ART (OR: 1.82, 95% CI:1.04–3.21) (53). Similar findings were observed In India, among 279, persons with high SFS score were 1.96 times more likely to be adherent (OR: 1.96; CI: 0.94–4.08) (55). Also, in a community-based study in 233 Nepalese living with HIV, negative emotional distance from family was associated with ART non-adherence (OR = 3.38, CI 1.28–8.91) (56). Therefore, social family support should be considered to be used to identify and prioritize those at high risk of non-adherence.

Body image dissatisfaction has been previously reported to have an adverse effect on ART adherence level (44). Generally, MSM, regardless of their HIV status, are more likely to have a high level of body dissatisfaction compared with heterosexuals (22, 57). Also, ART can cause body fat changes due to lipodystrophy (21, 44). Body image dissatisfaction may be an important indicator of non-adherence and cluster with other high-risk behaviors and psychosocial impacts that may benefit from interventions, including poor ART adherence (44, 45, 58), increased sexual behaviors that can transmit HIV (58), elevated levels of depressive symptoms (44, 58–60), increased methamphetamine use (61), and low self-esteem (62). In an Italian cohort study persons who had body tissue changes as a side effect of ART were almost five times more likely to be non-adherent (OR:4.67, 95% CI, 1.01–22.4) (63), and in an American cohort of 1,671 women self-perception of central fat gain was associated with a 1.5-fold increased odds of ART non-adherence (64). Finally, in a crosssectional study testing the impact of depression on the relationship between HAART adherence and body dissatisfaction in 124 HIV gay and heterosexual men, body dissatisfaction and depression independently predicted HAART non-adherence (44). Physical exercise might be a good intervention to improve depressive and anxiety symptoms and consequently adherence. A longitudinal study of three cohort studies done in the Netherlands among 7,076 participants showed that people who exercised were 1.47 times likely to recover from depression and anxiety (65).

There were several limitations of this study. Adherence was measured by self-report. Therefore, recall bias and social desirability bias may lead the participants to overreport their adherence level. Also, this crosssectional study was done within an intervention study with the aim of improving treatment adherence, and thus is not representative of those in standard care. Hence, level of adherence might be already enhanced compared with the general HIV population in the Philippines. The study population in SHIP clinic are also more highly educated than the general population in the Philippines and may not represent the general MSM population in the Philippines.

## Conclusions

This study was the first to assess the association between depression or other risk factors and ART non-adherence among MSM persons living with HIV in the Philippines. The findings of this study documented that depression is a relatively common symptom among MSM persons with HIV. Comprehensive mental health services beyond post-HIV testing counseling may increase adherence to ART and improve HIV treatment outcome. This can be done through routine screening for depression and anxiety symptoms using standardized validated tools like HADS, which can enable early identification of patients with mild or moderate symptoms. Simple interventions such as peer support, supervised by clinic program staff may help prevent the progression of symptoms and the development of complications. Patients with severe symptoms should be ideally referred to psychiatrists (if available) for further evaluation and care. Previous studies have shown that peer support can improve depressive symptoms more than ordinary care, as PLWHIV may prefer to share their experiences with others who are facing similar obstacles rather than reporting their psychological difficulties to psychiatrists or doctors (66, 67). In the SHIP clinic, doctors have subsequently started to conduct routine depression/anxiety screening using this tool, and they report that it enables patients to express their feelings and concerns and helps develop a stronger relationship between doctors and patients. Further prospective studies are needed to address the causal/reverse causal pathway between depression and non-adherence.

## Data Availability Statement

The original contributions presented in the study are included in the article/Supplementary Material, further inquiries can be directed to the corresponding author/s.

## Ethics Statement

The primary intervention study (CFL) was approved by the University of the Philippines Manila (reference number UPMREB 2016-265-01) London School of Hygiene and Tropical Medicine (reference number 11631). This nested cross-sectional study was approved by Nagasaki University, School of Tropical Medicine, and Global Health (reference number 42) The University of the Philippines Manila (reference number UPMREB 2017-453-01). The patients/participants provided their written informed consent to participate in this study.

## Author Contributions

HE, SC, CO'C, and ES designed the study. HE and KL implemented the study and collected the data. CO'C shared the data from the main cohort study. HE and SC conducted the statistical analysis, sample size calculation and wrote the manuscript. All authors contributed to and approved the final version of the manuscript for submission.

## Funding

This study was funded by Japan International Cooperation Agency through the ABE initiative scholarship to cover the salary of research nurses, ethical fees, and logistics.

## Conflict of Interest

The authors declare that the research was conducted in the absence of any commercial or financial relationships that could be construed as a potential conflict of interest.

## Publisher's Note

All claims expressed in this article are solely those of the authors and do not necessarily represent those of their affiliated organizations, or those of the publisher, the editors and the reviewers. Any product that may be evaluated in this article, or claim that may be made by its manufacturer, is not guaranteed or endorsed by the publisher.
