# Supplementary material for: Depression, Nutrition, and Adherence to Antiretroviral Therapy in Men Who Have Sex With Men in Manila, Philippines
Source: Front Public Health. 2021 Sep 21;9:644438. doi: 10.3389/fpubh.2021.644438 (PMC8490818; doi:10.3389/fpubh.2021.644438)
Supplement: Supplementary file 1 [file Table_1.DOCX]

**Supplementary Table 1 Socio-demographic and HIV-related behavioural/clinical factors and ART nonadherence**

|  |  |  |  |  |  |  |
| --- | --- | --- | --- | --- | --- | --- |
| **Risk factors** | | **Adherence** | | **Non-adherence** | | **X^2^  p-value** |
|  |  | **Frequency %** | | **Frequency %** | |  |
| **Age** | |  | |  | | 0.513 |
| Mean (95% CI) | | 32.05 (31.15 - 32.96) | | 31.20 (28.94 - 33.47) | |  |
| **Number of partners** | |  | |  | | 0.894 |
| Mean (95% CI) | | 3.05 (1.54 - 4.57) | | 3.33 (0.91 - 5.75) | |  |
| **CD4 count** | |  | |  | | 0.55 |
| Mean (95% CI) | | 493.21 (456.87 - 529.55) | | 529.73 (423.86 - 634.89) | |  |
| **Timepoint N (%)** | |  |  |  | |  |
| First week | | 2 (100.00%) | | 0 (0.00%) | | 0.740 |
| 12 weeks | | 3 (75.00%) | | 1 (25.00%) | |  |
| 24 weeks | | 40 (85.11%) | | 7 (14.89%) | |  |
| 48 weeks | | 124 (88.57%) | | 16 (11.43%) | |  |
| **sexual orientation** | |  |  |  |  | 0.6485 |
| Homosexual | | 106 (86.2%) | | 17 (13.8%) | |  |
| Bisexual | | 58 (90.6%) | | 5 (9.4%) | |  |
| Unknown | | 5 (83.3%) | | 1 (16.7%) | |  |
| **education level** | |  |  |  |  | 0.5172 |
| College or higher | | 141 (86.5%) | | 22 (13.5%) | |  |
| High School & vocational | | 8 (88.9%) | | 1 (11.1%) | |  |
| Unknown | | 20 (95.2%) | | 1 (4.8%) | |  |
| **employment** | |  |  |  |  | 0.6135 |
| employed | | 149 (87.1%) | | 22 (12.9%) | |  |
| unemployed | | 20 (90.9%) | | 2 (9.1%) | |  |
| Unknown | | 98 (86%) | | 16 (14.0%) | |  |
| **relationship status** | |  |  |  |  | 0.7174 |
| No relationship | | 98 (86%) | | 16 (14.0%) | |  |
| In relationship | | 43 (89.6%) | | 5 (10.4%) | |  |
| Unknown | | 28 (90.3%) | | 3 (9.7%) | |  |
| **disclosure status** | |  |  |  |  | 0.4155 |
| Disclosed | | 54 (91.5%) | | 5 (8.5%) | |  |
| Not Disclosed | | 82 (84.5%) | | 15 (15.5%) | |  |
| Unknown | | 33 (89.2%) | | 4 (10.8%) | |  |
| **civil status** | |  |  |  |  | 0.3016 |
| Single | | 159 (87.8%) | | 22 (12.2%) | |  |
| Married | | 5 (71.4%) | | 2 (28.6%) | |  |
| Unknown | | 5 (100%) | | 0 (0.00)% | |  |
| **active sexually** | |  |  |  |  | 0.9543 |
| Yes | | 83 (88.3%) | | 11 (11.7%) | |  |
| No | | 79 (86.8%) | | 12 (13.2%) | |  |
| Unknown | | 7 (87.5%) | | 1 (12.5%) | |  |
| **sex desire** | |  |  |  |  | 0.7301 |
| Men | | 140 (86.4%) | | 22 (13.6%) | |  |
| Both | | 27 (93.1%) | | 2 (6.9%) | |  |
| **using condom** | |  |  |  |  | 0.9543 |
| Yes | | 79 (86.8%) | | 12 (13.2%) | |  |
| No | | 83 (88.3%) | | 11 (11.7%) | |  |
| Unknown | | 7 (87.5%) | | 1 (12.5%) | |  |
| **frequency using condom** | |  |  |  |  | 0.1971 |
| Always/Most of the time | | 66 (85.7%) | | 11 (14.3%) | |  |
| Sometimes/Never | | 99 (90%) | | 11 (10%) | |  |
| Unknown | | 4 (66.7%) | | 2 (33.3%) | |  |
| **for commercial sex** | |  |  |  |  | 0.7531 |
| No | | 156 (87.2%) | | 23 (12.8%) | |  |
| Yes | | 10 (90.9%) | | 1 (9.1%) | |  |
| Unknown | | 3 (100%) | | 0 (0.00%) | |  |
| **using iv drugs** | |  |  |  |  | 0.6858 |
| No | | 139 (86.9%) | | 21 (13.1%) | |  |
| Yes | | 26 (89.7%) | | 3 (3.3%) | |  |
| Unknown | | 4 (100%) | | 0 (0.00%) | |  |
|  | |  |  |  |  |  |

Supplementary Table 2: Psychosocial, clinical and nutritional factors and depression

| **Risk Factor** | **Not depressed= 151** | | **Depressed=42** | | **P-value^¶1^** | **Crude OR (95%CI)** | | **P-value^¶2^** |
| --- | --- | --- | --- | --- | --- | --- | --- | --- |
| ***Social and psychological factors*** |  |  |  |  |  |  |  |  |
| **Age (years)** |  |  |  |  |  |  |  |  |
| Mean (95% CI) | 5.60 (4.99 - 6.22) | | 10.95 (9.81 - 12.10) | | 0.654 | 1.01 (0.96 - 1.073) | | 0.66 |
| **Anxiety HADS score** |  |  |  |  |  |  |  |  |
| Mean (95% CI) | 5.60 (4.99 - 6.22) | | 10.95 (9.81 - 12.10) | | **<0.001*** | 1.37 (1.24 - 1.52) | | <0.001 |
| **Self-esteem score** |  |  |  |  |  |  |  |  |
| Mean (95% CI) | 22.69 (21.88 - 23.50) | | 15.14 (13.58 - 16.61) | | **<0.001*** | 1.36 (1.23 - 1.51) | | <0.001 |
| **Social and family support score** |  |  |  |  |  |  |  |  |
| Mean (95% CI) | 55.13 (53.41 - 56.85) | | 40.67 (36.16 - 45.17) | | **<0.001*** | 1.10 (1.06 - 1.13) | | <0.001 |
| **Alcohol misuse score** |  |  |  |  |  |  |  |  |
| Mean (95% CI) | 3.50 (2.79 - 4.22) | | 4.24 (2.56 - 5.92) | | 0.3683 | 1.03 (0.96 - 1.11) | | 0.3686 |
| **Stigma score** |  |  |  |  |  |  |  |  |
| Mean (95% CI) | 111.12 (107.22 - 115.01) | | 132.67 (124.48 - 140.85) | | **<0.001*** | 1.04 (1.02 - 1.06) | | <0.001 |
| **BIQLI score** |  |  |  |  |  |  |  |  |
| Mean (95% CI) | 72.56 (71.06 - 74.05) | | 57.98 (53.90 - 62.05) | | **<0.001*** | 1.14 (1.09 - 1.19) | | <0.001 |
| ***Nutritional factors*^¶^*^3^*** |  |  |  |  |  |  |  |  |
| **BMI (kg/m2)** |  |  |  |  |  |  |  |  |
| Mean (95% CI) | 24.04 (23.53 - 24.56) | | 23.20 (22.19 - 24.21) | | 0.1300 | 0.92 (0.82 - 1.03) | | 0.131 |
| **MUAC (cm)** |  |  |  |  |  |  |  |  |
| Mean (95% CI) | 30.15 (29.51 - 30.80) | | 29.92 (28.76 - 31.08) | | 0.7379 | 0.98 (0.90 - 1.08) | | 0.7364 |
| ***Body composition*^¶^*^3^*** |  |  |  |  |  |  |  |  |
| **Waist/hip ratio** |  |  |  |  |  |  |  |  |
| Mean (95% CI) | 0.19 (0.90 - 0.92) | | 0.90 (0.89 - 0.92) | | 0.4382 | 0.12 (0.00 - 25.39) | | 0.4364 |
| **Total body fat (%)** |  |  |  |  |  |  |  |  |
| Mean (95% CI) | 18.01 (17.04 - 18.97) | | 15.68 (13.97 - 17.39) | | **0.0239*** | 0.94 (0.88 - 0.99) | | 0.026 |
| **Total lean body mass (%)** |  |  |  |  |  |  |  |  |
| Mean (95% CI) | 76.88 (75.22 - 78.54) | | 80.06 (78.44 - 81.67) | | **0.0544*** | 1.07 (1.01 - 1.14) | | 0.0303 |
| **Visceral fat (%)** |  |  |  |  |  |  |  |  |
| Mean (95% CI) | 5.26 (4.81 - 5.71) | | 4.36 (3.50 - 5.21) | | 0.0633* | 0.88 (0.78 - 1.01) | | 0.0654 |
| **Central fat mass (kg)** |  |  |  |  |  |  |  |  |
| Mean (95% CI) | 20.27 (19.07 - 21.47) | | 17.34 (15.29 - 19.39) | | **0.0219*** | 0.95 (0.90 - 0.99) | | 0.0239 |
| ***HIV/clinical factors*** |  |  |  |  |  |  |  |  |
| **Cd4 count (cells per μI) ^¶4^** |  |  |  |  |  |  |  |  |
| Mean (95% CI) | 509.86 (470.89 - 548.82) | | 455.59 (383.57 - 527.62) | | 0.1928 | 1.00 (0.99 - 1.05) | | 0.193 |

¶1 p-values from students t-test; 2 p-values from logistic regression; 3 Measurement of nutritional factors was missing for 1 person for BMI, 1 for MUAC and 2 for body composition measurements – all in the adherent group; 2 Four patients did not have data for CD4 count – all in adherent group
